# Impact of prior cancer history on the prognosis of extranodal NK/T-cell lymphoma

**DOI:** 10.1371/journal.pone.0311094

**Published:** 2024-10-16

**Authors:** Qian Wang, Tao Huang, Xudong Wei

**Affiliations:** 1 Department of Hematology and Oncology, The Affiliated Cancer Hospital of Zhengzhou University & Henan Cancer Hospital, Zhengzhou, Henan Province, PR China; 2 Department of Breast, The Affiliated Cancer Hospital of Zhengzhou University & Henan Cancer Hospital, Zhengzhou, Henan Province, PR China; UT Austin: The University of Texas at Austin, UNITED STATES OF AMERICA

## Abstract

Our goal was to assess the impact of prior cancer history on the prognosis of extranodal NK/T-cell lymphoma (ENKTCL). We searched the SEER database to retrospectively enroll patients with ENKTCL. The effects of cancer history on overall survival (OS) and disease-specific survival (DSS) were analyzed using the Cox model. A total of 691 patients were included, of whom 54 (7.8%) had prior histories of cancer. The most common solid malignancy was bone/soft tissue sarcoma. Most secondary ENKTCL cases occurred within 5–9 years following the first cancer diagnosis. Radiotherapy and chemotherapy had been administered to 45 and 40 patients, respectively, to treat their previous malignancies. Prior cancer history had little impact on DSS; however, the presence of prior solid cancer history, latency period of 10+ years, and prior administration of radiotherapy or chemotherapy significantly decreased OS. Prior cancer history had no effect on DSS, but survival compromised OS under specific circumstances.

## Introduction

Extranodal NK/T-cell lymphoma (ENKTCL) is a rare type of non-Hodgkin’s lymphoma (NHL) that accounts for approximately 2–10% of NHL cases [1]. Most cases originate from the nasal cavity and above the throat, although some originate outside the nose from areas such as the skin, gastrointestinal tract, and lungs [2]. ENKTCL is characterized by Epstein-Barr virus (EBV) infection, polymorphous lymphocyte infiltration centered around blood vessels, and tumor cell infiltration that damages blood vessels and causes necrosis [3,4]. The disease is known to be highly aggressive, with a high rate of treatment failure [5,6]. To achieve a satisfactory prognosis, multiple agent therapy is usually required [7,8].

Compared to treatments in the early 20th century, the 5-year overall survival (OS) of ENKTCL today can be as high as > 80% in patients with Ann Arbor stage I/II disease, and the median OS time has increased from < 1 year to > 3 years in patients with Ann Arbor stage III/IV disease [9]. These improvements in survival can be attributed largely to advances in radiation technology and chemotherapeutic drugs, but a standard treatment for this malignancy has still not been established. A number of ongoing clinical trials are aiming to determine the optimal management strategy for EKCTL, with the highest efficacy and least toxicity [10]—but one of the most prominent exclusion criteria in these trials is any prior history of cancer, in order to exclude its possible effect on survival.

The association between prior cancer history and secondary cancer survival has been well studied; however, conflicting results have been reported. Some have reported that no additional impact on OS is posed by prior cancer history [11,12], and that clinical trials should not refuse patients with prior cancer histories. Others support the idea that a prior cancer history adversely affects the prognosis of secondary cancer [13,14]. For example, patients with malignant melanoma and Merkel cell carcinoma who have a history of chronic lymphocytic leukemia have been found to have significantly worse OS than those without such history [15]. However, the impact of cancer history on the prognosis of ENKTCL has not yet been analyzed.

Therefore, this study aimed to clarify the effect of prior cancer history on ENKTCL and specifically assess whether the effect is determined by prior cancer type, latency period, and prior treatment method.

## Materials and methods

### Patient selection

All data were obtained from the National Cancer Institute’s SEER database (Incidence-SEER Research Data, 17 Registries, Nov 2022 Sub [2000–2020]). This database aims to reduce the cancer burden in the US population by providing publicly accessible cancer statistics. Data from a total of 1,180 patients with NK/T-cell lymphoma were retrospectively reviewed. The inclusion criteria were as follows: (1) the disease was primary or secondary to a previous primary cancer, with a latency period of > 2 months to exclude synchronous primary cancers [16], and (2) the patient received definite treatment for prior cancer and ENKTCL. Patients with nodal NK/T-cell lymphoma (n = 154), histories of multiple prior cancers (n = 8), and ENKCTL of unknown Ann Arbor stage (n = 327) were excluded. We extracted and analyzed data on demographic characteristics, histological grade, cancer stages, treatments, and follow-ups from the database. Ethical approval was not required for this study, as the data are publicly accessible.

### Variable definitions

To evaluate the potential effect of prior cancer history, three aspects were analyzed: type, treatment, and latency period. For type, the impacts of solid vs hematological malignancies were compared. For treatment, we only assessed the impact of radiotherapy and/or chemotherapy. For latency period, the patients were divided into two groups according to the median cutoff value. The effects of each level were described using hazard ratios (HRs) with 95% confidence intervals (CIs).

Ann Arbor staging was used to assess tumor severity. Prior radiotherapy referred to external-beam radiation therapy only. Latency was defined as the duration between the first cancer diagnosis and confirmation of ENKTCL. Ethnic background (Caucasian, African descent, or other) and marital status (married, single, or other) were also noted.

The primary outcome variables were OS and DSS. OS was calculated from the date of ENKTCL treatment to the date of death or final follow-up, and DSS was calculated from the treatment date to the date of cancer-related death or final follow-up.

### Statistical analyses

Differences in OS and DSS between patients with primary and secondary ENKTCL were evaluated using the Kaplan–Meier method. Univariate and multivariate Cox proportional hazards models were used to further explore the independent effects of prior cancer history on OS and DSS. All statistical analyses were performed using R version 3.4.3 (R Foundation for Statistical Computing, Vienna, Austria). Statistical significance was set at p < 0.05.

## Results

### Baseline data

Among the 691 patients enrolled, 339 (49.1%) were diagnosed before 2010, the mean age was 53 ± 16 years, there were 439 (63.5%) males and 252 (36.5%) females. The majority (70.6%) were Caucasian and 394 (57.0%) were married. A total of 47.5% had a one-year household income of $70,000 or more, and most (n = 526, 76.1%) lived in rural areas.

The Ann Arbor tumor stage was Ⅰ in 356 (51.5%), Ⅱ in 155 (22.4%), Ⅲ in 17 (2.5%), and Ⅳ in 163 (23.6%) of the patients. Treatment began within 1 month following diagnosis in 505 (73.1%) patients. Surgery, radiotherapy, and chemotherapy were used to treat the malignancies of 138 (20.0%), 417 (60.3%), and 482 (69.8%) of the patients, respectively. After a follow-up period with a mean time of 51 ± 59 months, 446 patients died—of which 352 were cancer-related deaths. The 5-year DSS and OS rates were 41% and 48%, respectively. Among cancer-unrelated deaths (n = 94), heart disease was found to be one of the most common causes (Table 1).

**Table 1:** Distribution of non-cancer-related death causes among patients with extranodal NK/T-cell lymphoma.

Cause	Number (%)
Diseases of Heart	18 (19.1%)
Diabetes Mellitus	5 (5.3%)
Pneumonia and Influenza	4 (4.3%)
Cerebrovascular Diseases	3 (3.2%)
Chronic Obstructive Pulmonary Disease and Allied Cond	3 (3.2%)
Other Infectious and Parasitic Diseases including HIV	3 (3.2%)
Accidents and Adverse Effects	2 (2.1%)
Lung and Bronchus	2 (2.1%)
Nephritis, Nephrotic Syndrome and Nephrosis	2 (2.1%)
Alzheimers	1 (1.1%)
Chronic Liver Disease and Cirrhosis	1 (1.1%)
Colon excluding Rectum	1 (1.1%)
Complications of Pregnancy, Childbirth, Puerperium	1 (1.1%)
Congenital Anomalies	1 (1.1%)
Liver	1 (1.1%)
Nasopharynx	1 (1.1%)
Other Diseases of Arteries, Arterioles, Capillaries	1 (1.1%)
Other Oral Cavity and Pharynx	1 (1.1%)
Prostate	1 (1.1%)
Rectum and Rectosigmoid Junction	1 (1.1%)
Septicemia	1 (1.1%)
Soft Tissue including Heart	1 (1.1%)
Stomach and Duodenal Ulcers	1 (1.1%)
Suicide and Self-Inflicted Injury	1 (1.1%)
Symptoms, Signs and Ill-Defined Conditions	1 (1.1%)
Tuberculosis	1 (1.1%)
Other Cause of Death	35 (37.3%)

A total of 54 (7.8%) of the patients had prior histories of cancer. Of these, 30 had solid malignancies, for which the most common form was bone/soft tissue sarcoma and the least common was gastric cancer. In the 24 patients who had hematological tumors, leukemia and lymphoma accounted for 50% each. Most (n = 31) secondary cases of ENKTCL occurred within 5–9 years after the first cancer diagnosis, although 10 cases developed 10 years later. Prior radiotherapy and chemotherapy had been administered to 45 and 40 patients, respectively (Table 2).

**Table 2:** Detailed information of 54 patients with a prior cancer history.

Feature	Number
Prior cancer type	
Solid (n = 30)	
Bone/soft tissue sarcoma	6
Colorectal cancer	5
Lung cancer	4
Renal cell carcinoma	4
Head and neck squamous cell carcinoma	3
Esophageal cancer	3
Central nerve system cancer	3
Gastric cancer	2
Hematologic (n = 24)	
Leukemia	12
Lymphoma	12
Latency (years)	
≤ 4	13
5–9	31
10+	10
Prior treatment	
Radiotherapy	45
Chemotherapy	40

### Univariate analysis

Factors such as age, primary cancer site, surgery, radiotherapy, and Ann Arbor stage were significantly associated with both DSS and OS (all p < 0.05). Chemotherapy (p < 0.001) and prior cancer history (p = 0.020) predicted worse OS but not DSS. Other variables, including sex, diagnosis year, ethnic background, marital status, household income, living area, and time to treatment did not have any impact on either DSS or OS (Table 3).

**Table 3:** Univariate analysis of predictors for disease specific survival (DSS) and overall survival (OS).

Variable	DSS	OS
Age	0.012	<0.001
Sex	0.607	0.843
Diagnosis year	0.232	0.150
Race	0.736	0.991
Marital status	0.412	0.755
Income	0.182	0.082
Area	0.741	0.615
Primary site	<0.001	<0.001
Surgery	<0.001	<0.001
Radiotherapy	<0.001	<0.001
Chemotherapy	0.148	<0.001
Time to treatment	0.208	0.894
Ann Arbor stage	<0.001	<0.001
Prior cancer history	0.429	0.020

### Multivariate analysis

In terms of DSS: an age of 60+ years had an HR of 1.56 (95% CI: 1.17–2.07), a non-nasal tumor location was associated with a 1.5-fold higher risk of death (95% CI: 1.21–1.87), the administration of radiotherapy provided better cancer control (HR: 0.54; 95% CI: 0.42–0.69), and Ann Arbor stage III/IV was associated with the highest possibility of cancer-related death (HR: 2.42; 95% CI: 1.92–3.04).

For OS: age ≥ 60 years increased overall risk of death (HR: 1.56; 95% CI: 1.20–2.01), a non-nasal primary site had an HR of 1.48 (95% CI: 1.22–1.79), both radiotherapy (HR: 0.58; 95% CI: 0.46–0.72) and chemotherapy (HR: 0.71; 95% CI: 0.58–0.88) were related to better survival possibility, and an Ann Arbor stage of Ⅲ or Ⅳ was the most significant prognosis factor—with an HR of 2.11 (95% CI: 1.71–2.61]. Prior cancer history (HR: 1.28; 95% CI: 0.92–1.77) did not alter the trend of overall death, and surgery was not associated with any additional survival benefit (Table 4).

**Table 4:** Multivariate analysis of predictors for disease specific survival (DSS) and overall survival (OS).

Variable	DSS		OS	
	p	HR[95%CI]	p	HR[95%CI]
Age				
≤39				
40–59	0.080	1.29[0.97–1.72]	0.171	1.20[0.93–1.55]
60+	0.002	1.56[1.17–2.07]	0.001	1.56[1.20–2.01]
Primary site				
Nasal				
Others	<0.001	1.51[1.21–1.87]	<0.001	1.48[1.22–1.79]
Surgery	0.485	0.89[0.65–1.23]	0.122	0.80[0.60–1.06]
Radiotherapy	<0.001	0.54[0.42–0.69]	<0.001	0.58[0.46–0.72]
Chemotherapy	-	-	<0.001	0.71[0.58–0.88]
Stage				
Ⅰ/Ⅱ				
Ⅲ/Ⅳ	<0.001	2.42[1.92–3.04]	<0.001	2.11[1.71–2.61]
Cancer history	-	-	0.138	1.28[0.92–1.77]

### Subgroup analysis

**Fig 1:**
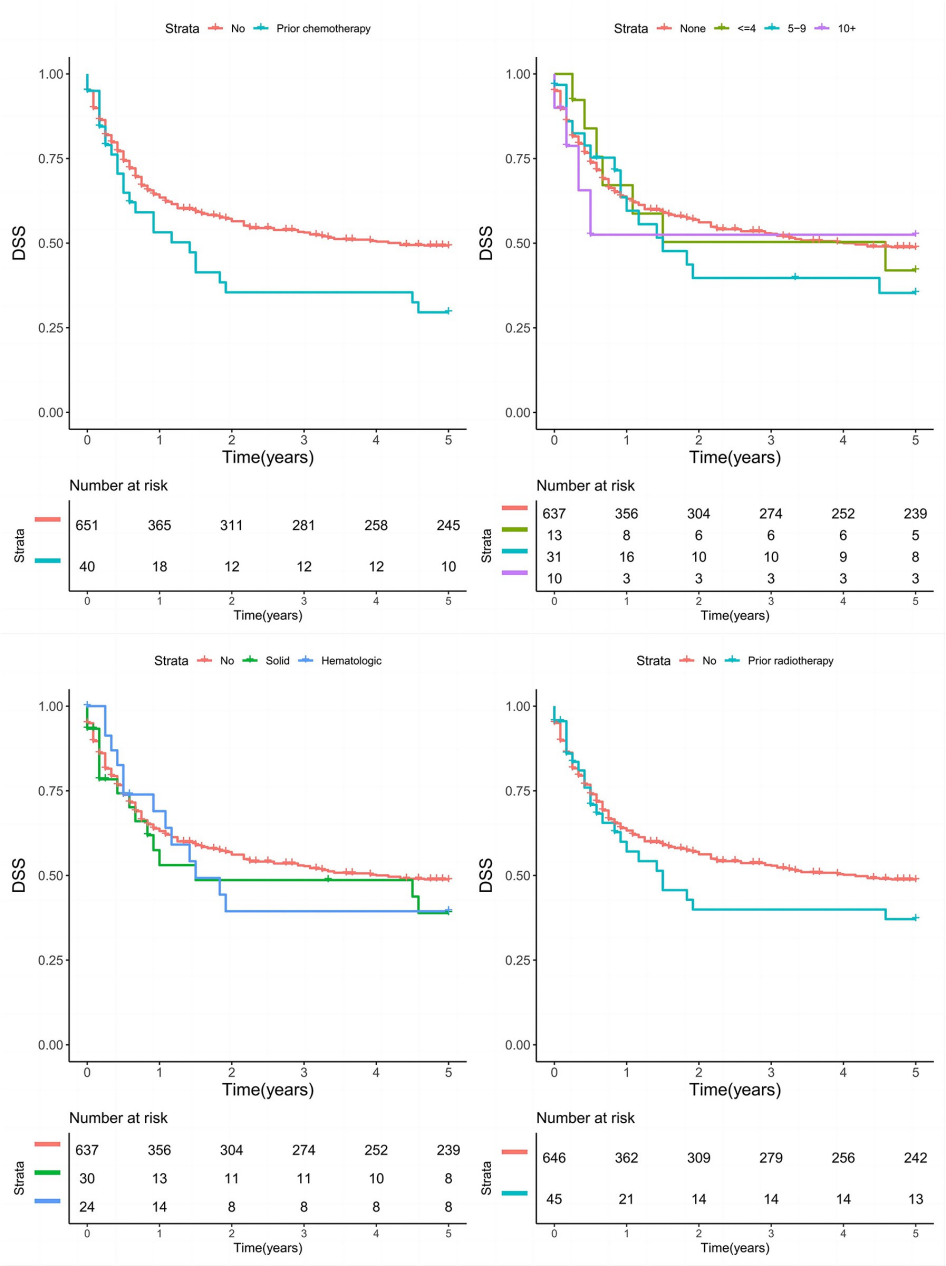
Disease specific survival (DSS) in patients with and without a cancer history classified by cancer type, latency period, prior radiotherapy, and prior chemotherapy.

For DSS, the three aspects of prior cancer history—including cancer type, latency, and prior treatment—showed only a limited impact on cancer-related death (Fig 1A–1D, all p > 0.05).

**Fig 2:**
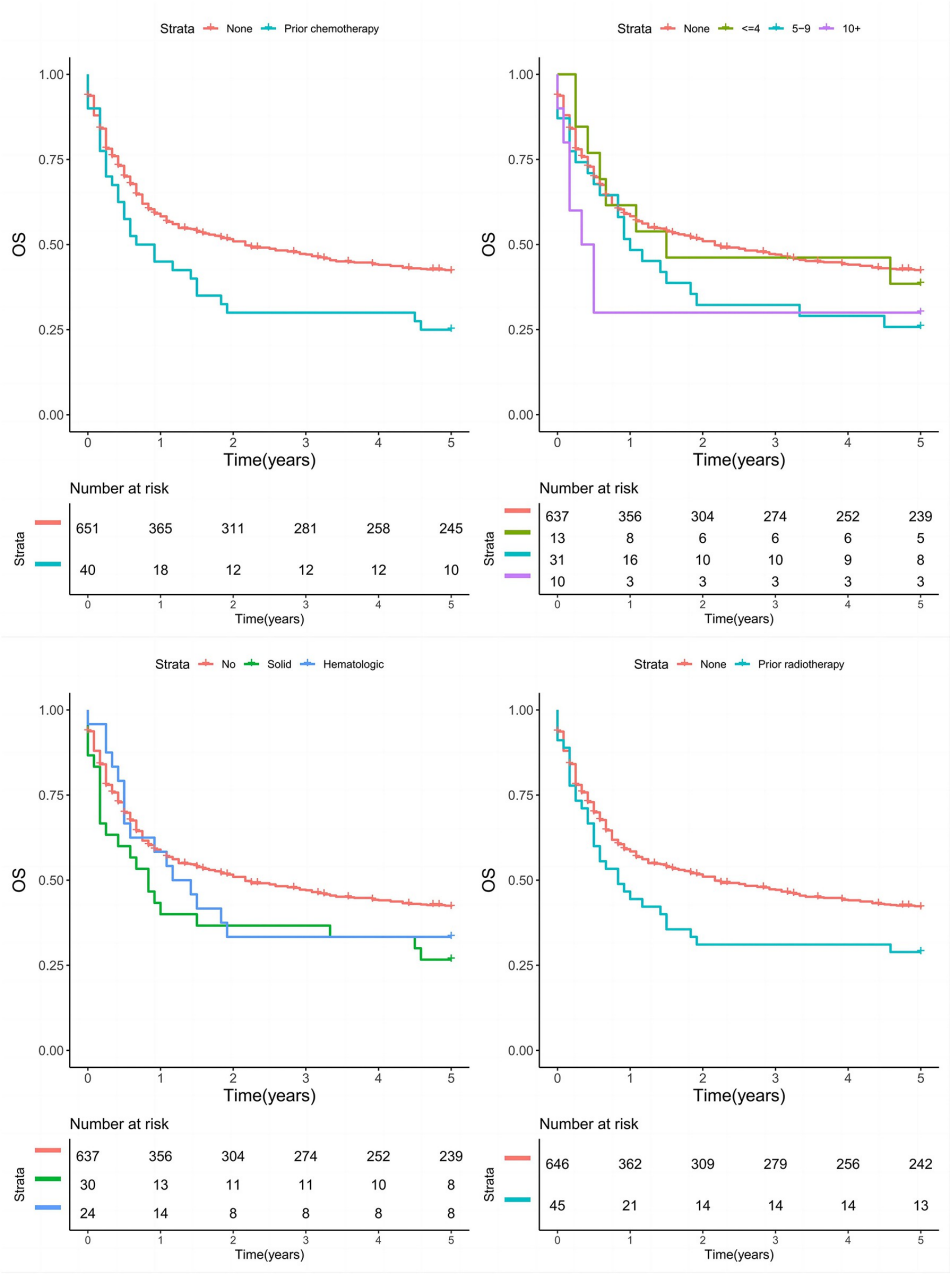
Overall survival (OS) in patients with and without a cancer history classified by cancer type, latency period, prior radiotherapy, and prior chemotherapy.

For OS, a prior history of solid (HR: 1.66; 95% CI: 1.11–2.46) rather than hematological cancer (HR: 1.20; 95% CI: 0.75–1.93) was related to poorer survival (Fig 2A). A latency period of 5–9 years (HR: 1.48; 95% CI: 0.99–2.21) tended to decrease OS, and a latency period of 10+ years (HR: 2.12; 95% CI: 1.13–3.98) was significantly linked to worse OS (Fig 2B). Prior treatment with radiotherapy (HR: 1.51; 95% CI: 1.08–2.12) and chemotherapy (HR: 1.52; 95% CI: 1.06–2.18) had comparably negative impacts on OS (Fig 2C and 2D; Table 5).

**Table 5:** Subgroup analysis of the impact of prior cancer history on disease specific survival (DSS) and overall survival (OS).

Subgroup	DSS		OS	
	p	HR[95%CI]	p	HR[95%CI]
Cancer type				
Solid	0.552	1.17[0.70–1.96]	0.013	1.66[1.11–2.46]
Hematologic	0.597	1.16[0.68–1.97]	0.445	1.20[0.75–1.93]
Latency (years)				
≤ 4	0.935	0.97[0.46–2.05]	0.992	1.00[0.52–1.93]
5–9	0.437	1.21[0.75–1.98]	0.059	1.48[0.99–2.21]
10+	0.507	1.35[0.56–3.26]	0.019	2.12[1.13–3.98]
Prior treatment				
Radiotherapy	0.265	1.26[0.84–1.89]	0.016	1.51[1.08–2.12]
Chemotherapy	0.035	1.54[1.03–2.30]	0.022	1.52[1.06–2.18]

## Discussion

The most important finding of this study was that secondary ENKTCL was relatively uncommon. A prior history of cancer was not found to affect the DSS of ENKCTL, irrespective of prior cancer type, latency time, or prior treatment. However, the presence of a prior solid cancer with a latency time of 10+ years, and the prior administration of radiotherapy or chemotherapy, significantly decreased OS. Our findings may provide grounds for changes to the design of inclusion criteria for ENKCTL-related clinical trials, potentially opening them to accepting patients with ENKTCL who do not possess these significant features, without a decrease in correctness.

Currently, owing to the assumption that prior cancer history might impact survival outcomes, patients who have been previously diagnosed with cancers have been excluded from most clinical trials. A substantial number of patients, particularly those with rare malignancies of low incidences, may be excluded from clinical trial enrollment because of these stringent eligibility criteria. This, in turn, can lead to limitations in the generalizability of results, trial duration extensions, and premature trial terminations [17]. The association between prior cancer history and secondary cancer has been studied extensively. Zhu et al. [18] analyzed the outcomes of 24,812 patients with larynx cancer, of whom 9.8% had prior histories of cancer. They found that, compared to those without a prior cancer history, having a prior cancer history served as a risk factor for OS—but a protective factor for DSS—and that the effect was determined by previous cancer type and latency time. On the one hand, the impact was limited to a latency period of > 12 months; on the other hand, a prior diagnosis of prostate cancer showed little impact on OS, but was a significant protective factor for DSS. A prior lung or breast cancer diagnosis, conversely, was associated with significantly lower OS, but not associated with DSS. Monsalve et al. [19] enrolled 821,323 patients with lung cancer and found that the 5-year OS in patients with prior cancer histories was slightly higher than those without, before adjustment. However, their adjusted analysis revealed that the impact of prior cancer history was extremely heterogeneous across different stages and treatment approaches. In their cohort, 51.4% of the patients fell into a subgroup in which prior cancer history appeared to compromise survival, 16.3% showed no significant difference, and 32.3% showed an association between prior cancer history and increased survival. Zhou et al. [20] performed a pan-cancer analysis in which two groups (PCI and PCS) were classified according to primary cancer site. Cancer developed in ~20% of the patients with prior cancer histories, compared to those without. The PCI group also had an inferior OS, while the PCS group had a similar OS. Laccetti et al. [21] reported that prior cancer history did not adversely affect the clinical outcomes of patients with advanced lung cancer, regardless of the stage or type of the prior cancer. Another study also suggested that the prognoses of patients with uterine papillary serous carcinoma were not affected by a prior history of breast cancer [22]. Conflicting results regarding the effect of a prior cancer history suggest that cancer type and latency time may represent the two important drivers, but this has never been evaluated for ENKTCL. To the best of our knowledge, this study is the first to analyze this topic. Compared to the studies mentioned above, the rate of prior cancer history was lower for the ENKTCL cases we analyzed, which might be explained by the fact that we found no shared pathogenic factors between the ENKTCL cases and any of the prior cancers. Interestingly, we also confirmed that cancer type and latency time tended to play a role in altering survival. Similar findings were also reported by others, where the negative effect of prior cancer history became apparent when the timing of prior cancer was > 24 months, and this effect was most significant when the timing exceeded 120 months, for nasopharyngeal carcinoma [23].

Radiotherapy and chemotherapy are essential parts of cancer treatment that may provide significant survival benefits, but may also cause secondary cancers. For example, the risk of secondary cancer increases to multiple extents across different radiation techniques in patients with breast cancer [24], and testosterone treatment is associated with a relatively higher incidence of breast cancer [25]. Considering the adverse reactions associated with these procedures, we deduced that prior treatment-related effects may have been more significant to our study than the prior cancers themselves. Unfortunately, the roles of such treatments with regard to long-term survival have rarely been studied. We noted that prior radiotherapy and chemotherapy significantly decreased both DSS and OS, which may be explained by the following aspects. First, prior treatments might weaken the immune system, which is the main weapon used to combat tumors. Second, prior treatments may limit the radiation doses and chemotherapy cycles prescribed to treat secondary cancers. Unfortunately, we could not find similar studies in the literature to corroborate these notions. The results of this study may help improve the determination of inclusion criteria in clinical trials, although further studies are needed to confirm our findings.

The study is subject to several limitations that must be acknowledged. First, the retrospective design of the study introduces inherent selection bias and reduces the ability to draw cause-and-effect conclusions. Additionally, the relatively small sample size limits the generalizability of the study findings and may limit the statistical power of the analyses. Second, the use of SEER database data may introduce potential biases and errors in the study results. Conflicting findings within the paper regarding the impact of prior cancer history on DSS and OS further complicate interpretation of the results. Third, the study suggests that prior treatments such as radiotherapy and chemotherapy may have negatively influenced survival outcomes. While these findings are significant, they require further research to confirm and validate their impact on patient prognosis.

## Conclusions

In summary, a prior history of cancer was not found to affect DSS, but the presence of a prior solid cancer, a latency period of 10+ years, and the prior administration of radiotherapy or chemotherapy significantly decreased OS. Patients with ENKTCL who do not have these significant features may be suitable for enrolment in clinical trials.

## Supporting information

S1 File (ZIP)
